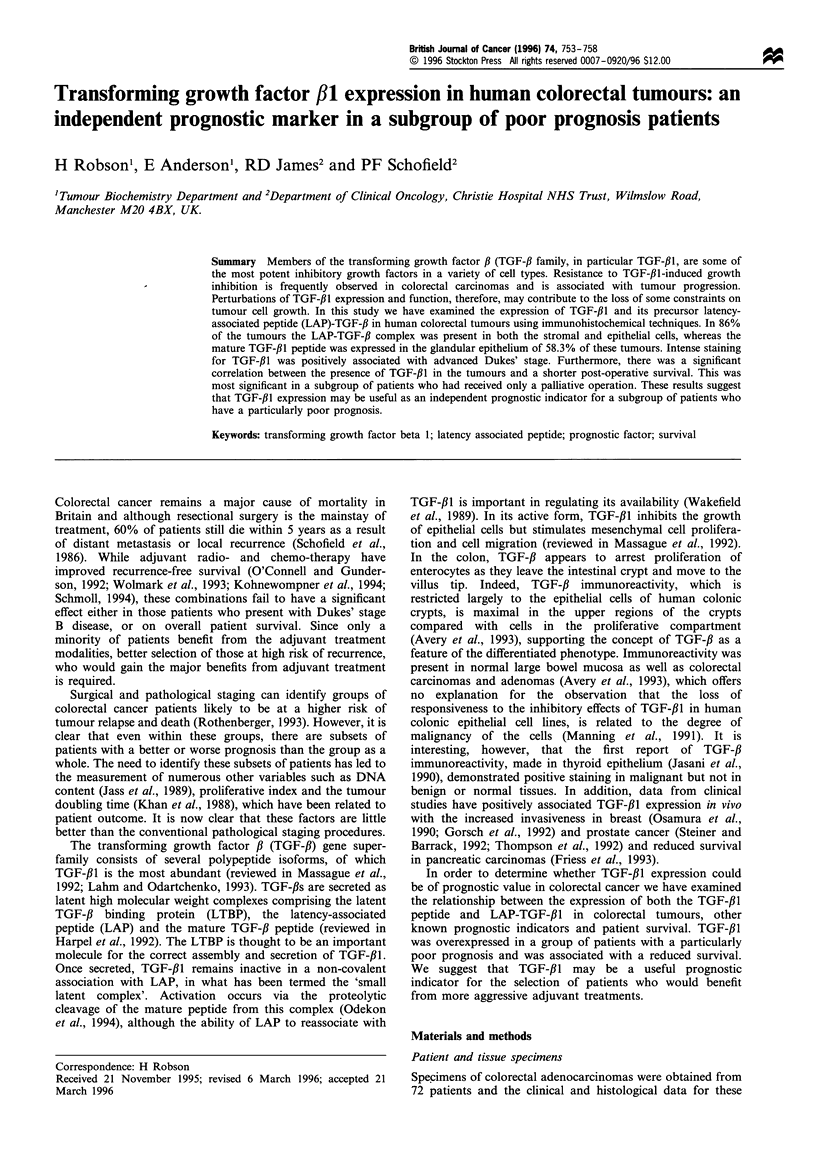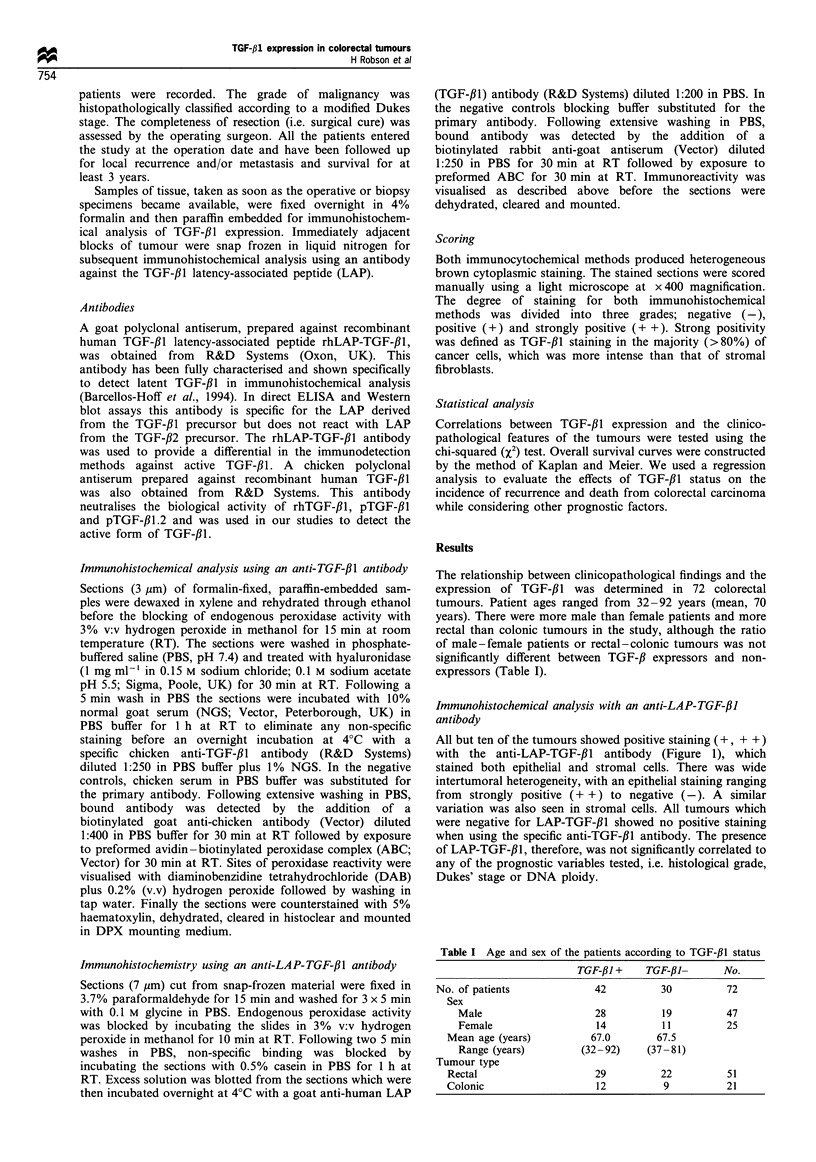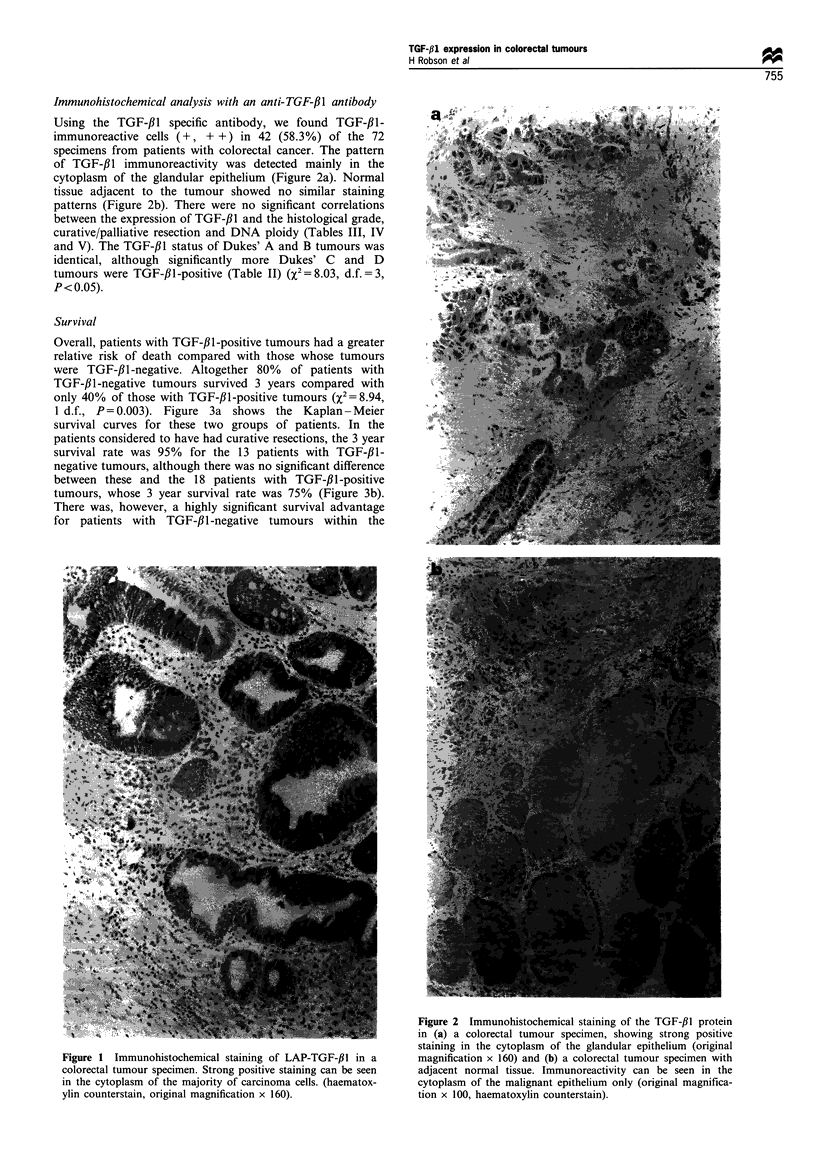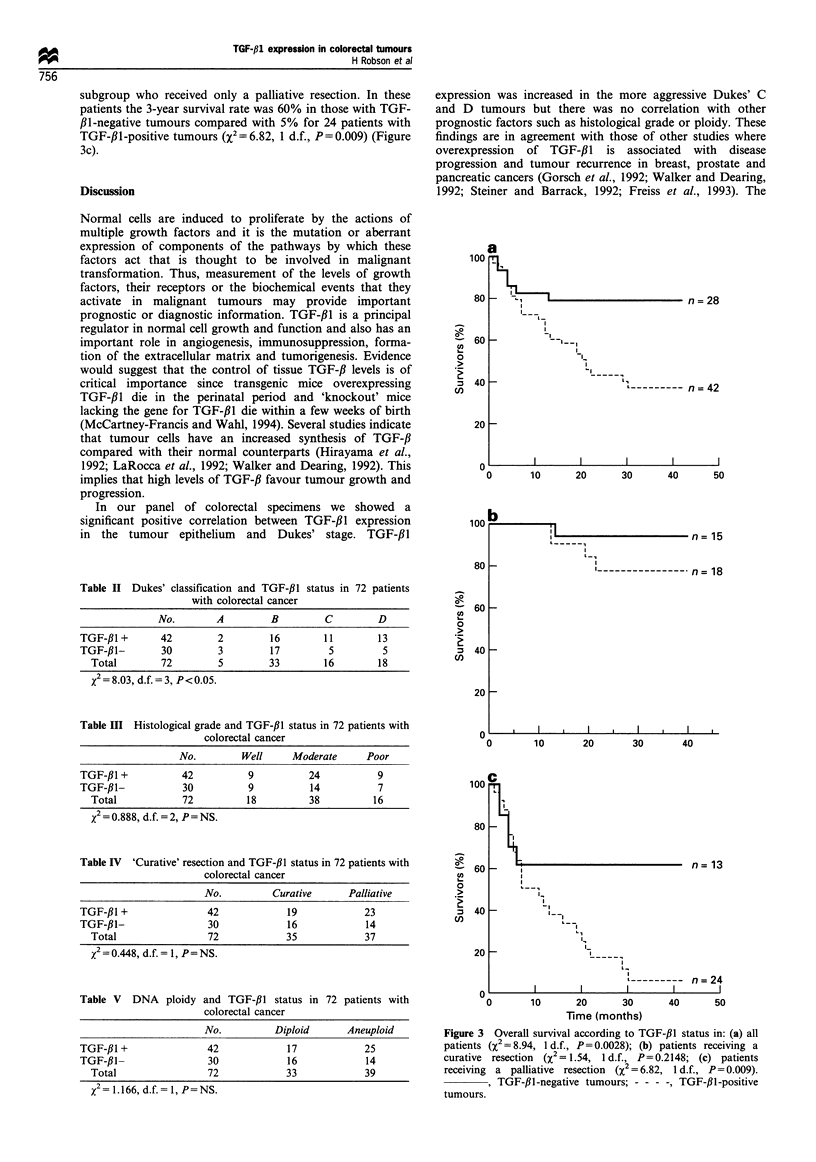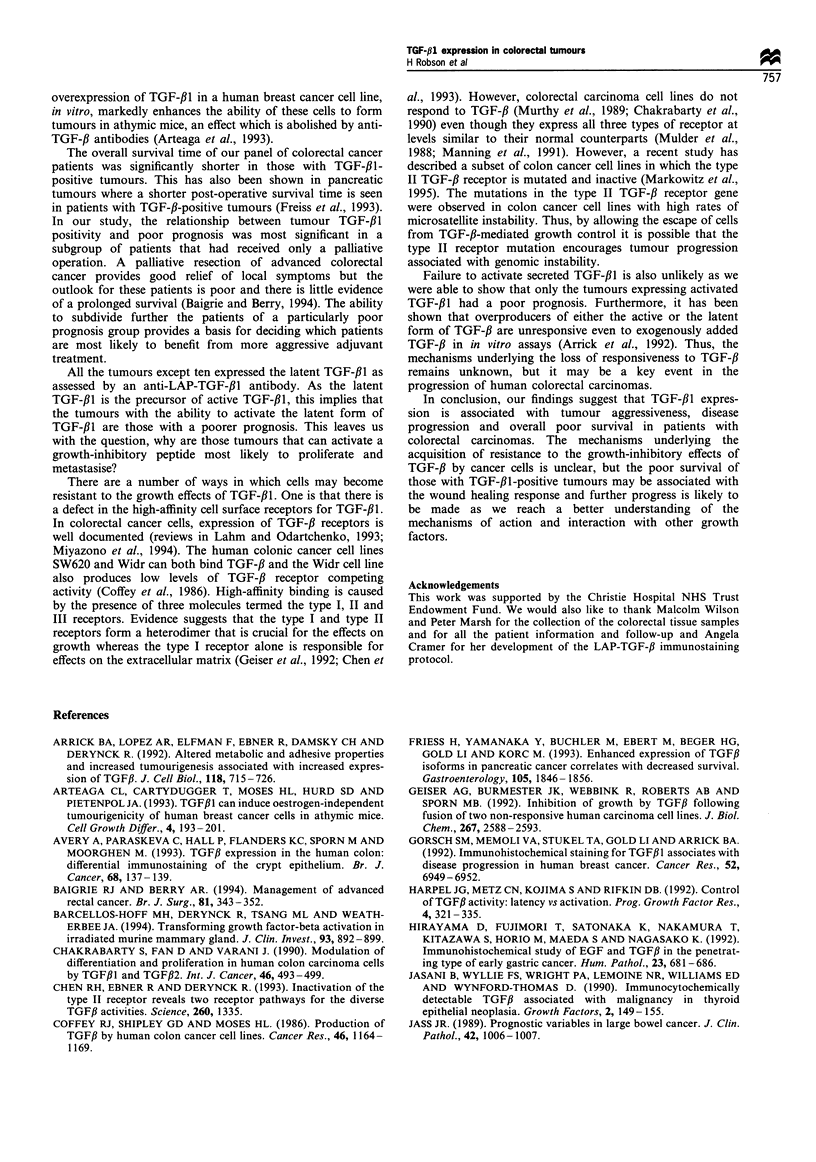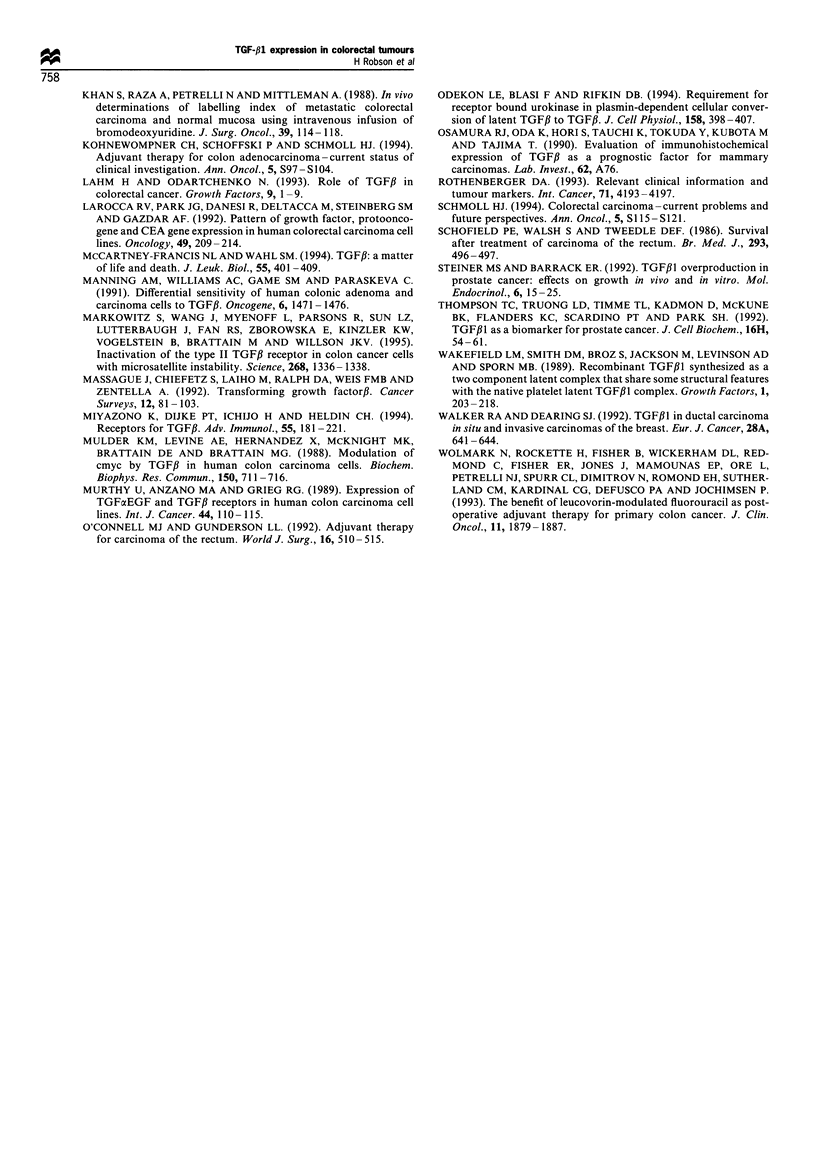# Transforming growth factor beta 1 expression in human colorectal tumours: an independent prognostic marker in a subgroup of poor prognosis patients.

**DOI:** 10.1038/bjc.1996.432

**Published:** 1996-09

**Authors:** H. Robson, E. Anderson, R. D. James, P. F. Schofield

**Affiliations:** Tumour Biochemistry Department, Christie Hospital NHS Trust, Manchester, UK.

## Abstract

**Images:**


British Journal of Cancer (1996) 74, 753-758
© 1996 Stockton Press All rights reserved 0007-0920/96 $12.00

### Transforming growth factor $\beta$1 expression in human colorectal tumours: an independent prognostic marker in a subgroup of poor prognosis patients

H Robson[1], E Anderson[1], RD James[2] and PF Schofield[2]

[1]*Tumour Biochemistry Department and* [2]*Department of Clinical Oncology, Christie Hospital NHS Trust, Wilmslow Road, Manchester M20 4BX, UK.*

**Summary** Members of the transforming growth factor $\beta$ (TGF-$\beta$ family, in particular TGF-$\beta$1, are some of the most potent inhibitory growth factors in a variety of cell types. Resistance to TGF-$\beta$1-induced growth inhibition is frequently observed in colorectal carcinomas and is associated with tumour progression. Perturbations of TGF-$\beta$1 expression and function, therefore, may contribute to the loss of some constraints on tumour cell growth. In this study we have examined the expression of TGF-$\beta$1 and its precursor latency-associated peptide (LAP)-TGF-$\beta$ in human colorectal tumours using immunohistochemical techniques. In 86% of the tumours the LAP-TGF-$\beta$ complex was present in both the stromal and epithelial cells, whereas the mature TGF-$\beta$1 peptide was expressed in the glandular epithelium of 58.3% of these tumours. Intense staining for TGF-$\beta$1 was positively associated with advanced Dukes' stage. Furthermore, there was a significant correlation between the presence of TGF-$\beta$1 in the tumours and a shorter post-operative survival. This was most significant in a subgroup of patients who had received only a palliative operation. These results suggest that TGF-$\beta$1 expression may be useful as an independent prognostic indicator for a subgroup of patients who have a particularly poor prognosis.

**Keywords:** transforming growth factor beta 1; latency associated peptide; prognostic factor; survival

Colorectal cancer remains a major cause of mortality in Britain and although resectional surgery is the mainstay of treatment, 60% of patients still die within 5 years as a result of distant metastasis or local recurrence (Schofield *et al.*, 1986). While adjuvant radio- and chemo-therapy have improved recurrence-free survival (O'Connell and Gunderson, 1992; Wolmark *et al.*, 1993; Kohnewompner *et al.*, 1994; Schmoll, 1994), these combinations fail to have a significant effect either in those patients who present with Dukes' stage B disease, or on overall patient survival. Since only a minority of patients benefit from the adjuvant treatment modalities, better selection of those at high risk of recurrence, who would gain the major benefits from adjuvant treatment is required.

Surgical and pathological staging can identify groups of colorectal cancer patients likely to be at a higher risk of tumour relapse and death (Rothenberger, 1993). However, it is clear that even within these groups, there are subsets of patients with a better or worse prognosis than the group as a whole. The need to identify these subsets of patients has led to the measurement of numerous other variables such as DNA content (Jass *et al.*, 1989), proliferative index and the tumour doubling time (Khan *et al.*, 1988), which have been related to patient outcome. It is now clear that these factors are little better than the conventional pathological staging procedures.

The transforming growth factor $\beta$ (TGF-$\beta$) gene superfamily consists of several polypeptide isoforms, of which TGF-$\beta$1 is the most abundant (reviewed in Massague *et al.*, 1992; Lahm and Odartchenko, 1993). TGF-$\beta$s are secreted as latent high molecular weight complexes comprising the latent TGF-$\beta$ binding protein (LTBP), the latency-associated peptide (LAP) and the mature TGF-$\beta$ peptide (reviewed in Harpel *et al.*, 1992). The LTBP is thought to be an important molecule for the correct assembly and secretion of TGF-$\beta$1. Once secreted, TGF-$\beta$1 remains inactive in a non-covalent association with LAP, in what has been termed the 'small latent complex'. Activation occurs via the proteolytic cleavage of the mature peptide from this complex (Odekon *et al.*, 1994), although the ability of LAP to reassociate with TGF-$\beta$1 is important in regulating its availability (Wakefield *et al.*, 1989). In its active form, TGF-$\beta$1 inhibits the growth of epithelial cells but stimulates mesenchymal cell proliferation and cell migration (reviewed in Massague *et al.*, 1992). In the colon, TGF-$\beta$ appears to arrest proliferation of enterocytes as they leave the intestinal crypt and move to the villus tip. Indeed, TGF-$\beta$ immunoreactivity, which is restricted largely to the epithelial cells of human colonic crypts, is maximal in the upper regions of the crypts compared with cells in the proliferative compartment (Avery *et al.*, 1993), supporting the concept of TGF-$\beta$ as a feature of the differentiated phenotype. Immunoreactivity was present in normal large bowel mucosa as well as colorectal carcinomas and adenomas (Avery *et al.*, 1993), which offers no explanation for the observation that the loss of responsiveness to the inhibitory effects of TGF-$\beta$1 in human colonic epithelial cell lines, is related to the degree of malignancy of the cells (Manning *et al.*, 1991). It is interesting, however, that the first report of TGF-$\beta$ immunoreactivity, made in thyroid epithelium (Jasani *et al.*, 1990), demonstrated positive staining in malignant but not in benign or normal tissues. In addition, data from clinical studies have positively associated TGF-$\beta$1 expression *in vivo* with the increased invasiveness in breast (Osamura *et al.*, 1990; Gorsch *et al.*, 1992) and prostate cancer (Steiner and Barrack, 1992; Thompson *et al.*, 1992) and reduced survival in pancreatic carcinomas (Friess *et al.*, 1993).

In order to determine whether TGF-$\beta$1 expression could be of prognostic value in colorectal cancer we have examined the relationship between the expression of both the TGF-$\beta$1 peptide and LAP-TGF-$\beta$1 in colorectal tumours, other known prognostic indicators and patient survival. TGF-$\beta$1 was overexpressed in a group of patients with a particularly poor prognosis and was associated with a reduced survival. We suggest that TGF-$\beta$1 may be a useful prognostic indicator for the selection of patients who would benefit from more aggressive adjuvant treatments.

### Materials and methods

### *Patient and tissue specimens*

Specimens of colorectal adenocarcinomas were obtained from 72 patients and the clinical and histological data for these

Correspondence: H Robson
Received 21 November 1995; revised 6 March 1996; accepted 21 March 1996

patients were recorded. The grade of malignancy was histopathologically classified according to a modified Dukes stage. The completeness of resection (i.e. surgical cure) was assessed by the operating surgeon. All the patients entered the study at the operation date and have been followed up for local recurrence and/or metastasis and survival for at least 3 years.

Samples of tissue, taken as soon as the operative or biopsy specimens became available, were fixed overnight in 4% formalin and then paraffin embedded for immunohistochemical analysis of TGF-$\beta$1 expression. Immediately adjacent blocks of tumour were snap frozen in liquid nitrogen for subsequent immunohistochemical analysis using an antibody against the TGF-$\beta$1 latency-associated peptide (LAP).

*Antibodies*

A goat polyclonal antiserum, prepared against recombinant human TGF-$\beta$1 latency-associated peptide rhLAP-TGF-$\beta$1, was obtained from R&D Systems (Oxon, UK). This antibody has been fully characterised and shown specifically to detect latent TGF-$\beta$1 in immunohistochemical analysis (Barcellos-Hoff *et al.*, 1994). In direct ELISA and Western blot assays this antibody is specific for the LAP derived from the TGF-$\beta$1 precursor but does not react with LAP from the TGF-$\beta$2 precursor. The rhLAP-TGF-$\beta$1 antibody was used to provide a differential in the immunodetection methods against active TGF-$\beta$1. A chicken polyclonal antiserum prepared against recombinant human TGF-$\beta$1 was also obtained from R&D Systems. This antibody neutralises the biological activity of rhTGF-$\beta$1, pTGF-$\beta$1 and pTGF-$\beta$1.2 and was used in our studies to detect the active form of TGF-$\beta$1.

*Immunohistochemical analysis using an anti-TGF-$\beta$1 antibody*

Sections (3 $\mu$m) of formalin-fixed, paraffin-embedded samples were dewaxed in xylene and rehydrated through ethanol before the blocking of endogenous peroxidase activity with 3% v:v hydrogen peroxide in methanol for 15 min at room temperature (RT). The sections were washed in phosphate-buffered saline (PBS, pH 7.4) and treated with hyaluronidase (1 mg $ml^{-1}$ in 0.15 M sodium chloride; 0.1 M sodium acetate pH 5.5; Sigma, Poole, UK) for 30 min at RT. Following a 5 min wash in PBS the sections were incubated with 10% normal goat serum (NGS; Vector, Peterborough, UK) in PBS buffer for 1 h at RT to eliminate any non-specific staining before an overnight incubation at 4°C with a specific chicken anti-TGF-$\beta$1 antibody (R&D Systems) diluted 1:250 in PBS buffer plus 1% NGS. In the negative controls, chicken serum in PBS buffer was substituted for the primary antibody. Following extensive washing in PBS, bound antibody was detected by the addition of a biotinylated goat anti-chicken antibody (Vector) diluted 1:400 in PBS buffer for 30 min at RT followed by exposure to preformed avidin–biotinylated peroxidase complex (ABC; Vector) for 30 min at RT. Sites of peroxidase reactivity were visualised with diaminobenzidine tetrahydrochloride (DAB) plus 0.2% (v.v) hydrogen peroxide followed by washing in tap water. Finally the sections were counterstained with 5% haematoxylin, dehydrated, cleared in histoclear and mounted in DPX mounting medium.

*Immunohistochemistry using an anti-LAP-TGF-$\beta$1 antibody*

Sections (7 $\mu$m) cut from snap-frozen material were fixed in 3.7% paraformaldehyde for 15 min and washed for 3 × 5 min with 0.1 M glycine in PBS. Endogenous peroxidase activity was blocked by incubating the slides in 3% v:v hydrogen peroxide in methanol for 10 min at RT. Following two 5 min washes in PBS, non-specific binding was blocked by incubating the sections with 0.5% casein in PBS for 1 h at RT. Excess solution was blotted from the sections which were then incubated overnight at 4°C with a goat anti-human LAP (TGF-$\beta$1) antibody (R&D Systems) diluted 1:200 in PBS. In the negative controls blocking buffer substituted for the primary antibody. Following extensive washing in PBS, bound antibody was detected by the addition of a biotinylated rabbit anti-goat antiserum (Vector) diluted 1:250 in PBS for 30 min at RT followed by exposure to preformed ABC for 30 min at RT. Immunoreactivity was visualised as described above before the sections were dehydrated, cleared and mounted.

*Scoring*

Both immunocytochemical methods produced heterogeneous brown cytoplasmic staining. The stained sections were scored manually using a light microscope at ×400 magnification. The degree of staining for both immunohistochemical methods was divided into three grades; negative (−), positive (+) and strongly positive (+ +). Strong positivity was defined as TGF-$\beta$1 staining in the majority (>80%) of cancer cells, which was more intense than that of stromal fibroblasts.

*Statistical analysis*

Correlations between TGF-$\beta$1 expression and the clinicopathological features of the tumours were tested using the chi-squared ($\chi^2$) test. Overall survival curves were constructed by the method of Kaplan and Meier. We used a regression analysis to evaluate the effects of TGF-$\beta$1 status on the incidence of recurrence and death from colorectal carcinoma while considering other prognostic factors.

### Results

The relationship between clinicopathological findings and the expression of TGF-$\beta$1 was determined in 72 colorectal tumours. Patient ages ranged from 32–92 years (mean, 70 years). There were more male than female patients and more rectal than colonic tumours in the study, although the ratio of male–female patients or rectal–colonic tumours was not significantly different between TGF-$\beta$ expressors and non-expressors (Table I).

*Immunohistochemical analysis with an anti-LAP-TGF-$\beta$1 antibody*

All but ten of the tumours showed positive staining (+, + +) with the anti-LAP-TGF-$\beta$1 antibody (Figure 1), which stained both epithelial and stromal cells. There was wide intertumoral heterogeneity, with an epithelial staining ranging from strongly positive (+ +) to negative (−). A similar variation was also seen in stromal cells. All tumours which were negative for LAP-TGF-$\beta$1 showed no positive staining when using the specific anti-TGF-$\beta$1 antibody. The presence of LAP-TGF-$\beta$1, therefore, was not significantly correlated to any of the prognostic variables tested, i.e. histological grade, Dukes' stage or DNA ploidy.

**Table I** Age and sex of the patients according to TGF-$\beta$1 status

| | *TGF-$\beta$1+* | *TGF-$\beta$1−* | *No.* |
|---|---|---|---|
| No. of patients | 42 | 30 | 72 |
| Sex | | | |
| Male | 28 | 19 | 47 |
| Female | 14 | 11 | 25 |
| Mean age (years) | 67.0 | 67.5 | |
| Range (years) | (32–92) | (37–81) | |
| Tumour type | | | |
| Rectal | 29 | 22 | 51 |
| Colonic | 12 | 9 | 21 |

*Immunohistochemical analysis with an anti-TGF-β1 antibody*

Using the TGF-β1 specific antibody, we found TGF-β1-immunoreactive cells (+, ++) in 42 (58.3%) of the 72 specimens from patients with colorectal cancer. The pattern of TGF-β1 immunoreactivity was detected mainly in the cytoplasm of the glandular epithelium (Figure 2a). Normal tissue adjacent to the tumour showed no similar staining patterns (Figure 2b). There were no significant correlations between the expression of TGF-β1 and the histological grade, curative/palliative resection and DNA ploidy (Tables III, IV and V). The TGF-β1 status of Dukes' A and B tumours was identical, although significantly more Dukes' C and D tumours were TGF-β1-positive (Table II) ($\chi^2 = 8.03$, d.f. = 3, $P < 0.05$).

*Survival*

Overall, patients with TGF-β1-positive tumours had a greater relative risk of death compared with those whose tumours were TGF-β1-negative. Altogether 80% of patients with TGF-β1-negative tumours survived 3 years compared with only 40% of those with TGF-β1-positive tumours ($\chi^2 = 8.94$, 1 d.f., $P = 0.003$). Figure 3a shows the Kaplan–Meier survival curves for these two groups of patients. In the patients considered to have had curative resections, the 3 year survival rate was 95% for the 13 patients with TGF-β1-negative tumours, although there was no significant difference between these and the 18 patients with TGF-β1-positive tumours, whose 3 year survival rate was 75% (Figure 3b). There was, however, a highly significant survival advantage for patients with TGF-β1-negative tumours within the

**Figure 1** Immunohistochemical staining of LAP-TGF-β1 in a colorectal tumour specimen. Strong positive staining can be seen in the cytoplasm of the majority of carcinoma cells. (haematoxylin counterstain, original magnification × 160).

**Figure 2** Immunohistochemical staining of the TGF-β1 protein in (**a**) a colorectal tumour specimen, showing strong positive staining in the cytoplasm of the glandular epithelium (original magnification × 160) and (**b**) a colorectal tumour specimen with adjacent normal tissue. Immunoreactivity can be seen in the cytoplasm of the malignant epithelium only (original magnification × 100, haematoxylin counterstain).

subgroup who received only a palliative resection. In these patients the 3-year survival rate was 60% in those with TGF-β1-negative tumours compared with 5% for 24 patients with TGF-β1-positive tumours ($\chi^2=6.82$, 1 d.f., $P=0.009$) (Figure 3c).

### Discussion

Normal cells are induced to proliferate by the actions of multiple growth factors and it is the mutation or aberrant expression of components of the pathways by which these factors act that is thought to be involved in malignant transformation. Thus, measurement of the levels of growth factors, their receptors or the biochemical events that they activate in malignant tumours may provide important prognostic or diagnostic information. TGF-β1 is a principal regulator in normal cell growth and function and also has an important role in angiogenesis, immunosuppression, formation of the extracellular matrix and tumorigenesis. Evidence would suggest that the control of tissue TGF-β levels is of critical importance since transgenic mice overexpressing TGF-β1 die in the perinatal period and 'knockout' mice lacking the gene for TGF-β1 die within a few weeks of birth (McCartney-Francis and Wahl, 1994). Several studies indicate that tumour cells have an increased synthesis of TGF-β compared with their normal counterparts (Hirayama et al., 1992; LaRocca et al., 1992; Walker and Dearing, 1992). This implies that high levels of TGF-β favour tumour growth and progression.

In our panel of colorectal specimens we showed a significant positive correlation between TGF-β1 expression in the tumour epithelium and Dukes' stage. TGF-β1 expression was increased in the more aggressive Dukes' C and D tumours but there was no correlation with other prognostic factors such as histological grade or ploidy. These findings are in agreement with those of other studies where overexpression of TGF-β1 is associated with disease progression and tumour recurrence in breast, prostate and pancreatic cancers (Gorsch et al., 1992; Walker and Dearing, 1992; Steiner and Barrack, 1992; Freiss et al., 1993). The

**Table II** Dukes' classification and TGF-β1 status in 72 patients with colorectal cancer

| | No. | A | B | C | D |
|---|---|---|---|---|---|
| TGF-β1+ | 42 | 2 | 16 | 11 | 13 |
| TGF-β1− | 30 | 3 | 17 | 5 | 5 |
| Total | 72 | 5 | 33 | 16 | 18 |

$\chi^2=8.03$, d.f. = 3, $P<0.05$.

**Table III** Histological grade and TGF-β1 status in 72 patients with colorectal cancer

| | No. | Well | Moderate | Poor |
|---|---|---|---|---|
| TGF-β1+ | 42 | 9 | 24 | 9 |
| TGF-β1− | 30 | 9 | 14 | 7 |
| Total | 72 | 18 | 38 | 16 |

$\chi^2=0.888$, d.f. = 2, $P$ = NS.

**Table IV** 'Curative' resection and TGF-β1 status in 72 patients with colorectal cancer

| | No. | Curative | Palliative |
|---|---|---|---|
| TGF-β1+ | 42 | 19 | 23 |
| TGF-β1− | 30 | 16 | 14 |
| Total | 72 | 35 | 37 |

$\chi^2=0.448$, d.f. = 1, $P$ = NS.

**Table V** DNA ploidy and TGF-β1 status in 72 patients with colorectal cancer

| | No. | Diploid | Aneuploid |
|---|---|---|---|
| TGF-β1+ | 42 | 17 | 25 |
| TGF-β1− | 30 | 16 | 14 |
| Total | 72 | 33 | 39 |

$\chi^2=1.166$, d.f. = 1, $P$ = NS.

**Figure 3** Overall survival according to TGF-β1 status in: (**a**) all patients ($\chi^2=8.94$, 1 d.f., $P=0.0028$); (**b**) patients receiving a curative resection ($\chi^2=1.54$, 1 d.f., $P=0.2148$); (**c**) patients receiving a palliative resection ($\chi^2=6.82$, 1 d.f., $P=0.009$). ———, TGF-β1-negative tumours; - - - -, TGF-β1-positive tumours.

overexpression of TGF-β1 in a human breast cancer cell line, *in vitro*, markedly enhances the ability of these cells to form tumours in athymic mice, an effect which is abolished by anti-TGF-β antibodies (Arteaga *et al*., 1993).

The overall survival time of our panel of colorectal cancer patients was significantly shorter in those with TGF-β1-positive tumours. This has also been shown in pancreatic tumours where a shorter post-operative survival time is seen in patients with TGF-β-positive tumours (Freiss *et al*., 1993). In our study, the relationship between tumour TGF-β1 positivity and poor prognosis was most significant in a subgroup of patients that had received only a palliative operation. A palliative resection of advanced colorectal cancer provides good relief of local symptoms but the outlook for these patients is poor and there is little evidence of a prolonged survival (Baigrie and Berry, 1994). The ability to subdivide further the patients of a particularly poor prognosis group provides a basis for deciding which patients are most likely to benefit from more aggressive adjuvant treatment.

All the tumours except ten expressed the latent TGF-β1 as assessed by an anti-LAP-TGF-β1 antibody. As the latent TGF-β1 is the precursor of active TGF-β1, this implies that the tumours with the ability to activate the latent form of TGF-β1 are those with a poorer prognosis. This leaves us with the question, why are those tumours that can activate a growth-inhibitory peptide most likely to proliferate and metastasise?

There are a number of ways in which cells may become resistant to the growth effects of TGF-β1. One is that there is a defect in the high-affinity cell surface receptors for TGF-β1. In colorectal cancer cells, expression of TGF-β receptors is well documented (reviews in Lahm and Odartchenko, 1993; Miyazono *et al*., 1994). The human colonic cancer cell lines SW620 and Widr can both bind TGF-β and the Widr cell line also produces low levels of TGF-β receptor competing activity (Coffey *et al*., 1986). High-affinity binding is caused by the presence of three molecules termed the type I, II and III receptors. Evidence suggests that the type I and type II receptors form a heterodimer that is crucial for the effects on growth whereas the type I receptor alone is responsible for effects on the extracellular matrix (Geiser *et al*., 1992; Chen *et al*., 1993). However, colorectal carcinoma cell lines do not respond to TGF-β (Murthy *et al*., 1989; Chakrabarty *et al*., 1990) even though they express all three types of receptor at levels similar to their normal counterparts (Mulder *et al*., 1988; Manning *et al*., 1991). However, a recent study has described a subset of colon cancer cell lines in which the type II TGF-β receptor is mutated and inactive (Markowitz *et al*., 1995). The mutations in the type II TGF-β receptor gene were observed in colon cancer cell lines with high rates of microsatellite instability. Thus, by allowing the escape of cells from TGF-β-mediated growth control it is possible that the type II receptor mutation encourages tumour progression associated with genomic instability.

Failure to activate secreted TGF-β1 is also unlikely as we were able to show that only the tumours expressing activated TGF-β1 had a poor prognosis. Furthermore, it has been shown that overproducers of either the active or the latent form of TGF-β are unresponsive even to exogenously added TGF-β in *in vitro* assays (Arrick *et al*., 1992). Thus, the mechanisms underlying the loss of responsiveness to TGF-β remains unknown, but it may be a key event in the progression of human colorectal carcinomas.

In conclusion, our findings suggest that TGF-β1 expression is associated with tumour aggressiveness, disease progression and overall poor survival in patients with colorectal carcinomas. The mechanisms underlying the acquisition of resistance to the growth-inhibitory effects of TGF-β by cancer cells is unclear, but the poor survival of those with TGF-β1-positive tumours may be associated with the wound healing response and further progress is likely to be made as we reach a better understanding of the mechanisms of action and interaction with other growth factors.

**Acknowledgements**
This work was supported by the Christie Hospital NHS Trust Endowment Fund. We would also like to thank Malcolm Wilson and Peter Marsh for the collection of the colorectal tissue samples and for all the patient information and follow-up and Angela Cramer for her development of the LAP-TGF-β immunostaining protocol.

### References

ARRICK BA, LOPEZ AR, ELFMAN F, EBNER R, DAMSKY CH AND DERYNCK R. (1992). Altered metabolic and adhesive properties and increased tumourigenesis associated with increased expression of TGFβ. *J. Cell Biol*., **118,** 715–726.

ARTEAGA CL, CARTYDUGGER T, MOSES HL, HURD SD AND PIETENPOL JA. (1993). TGFβ1 can induce oestrogen-independent tumourigenicity of human breast cancer cells in athymic mice. *Cell Growth Differ*., **4,** 193–201.

AVERY A, PARASKEVA C, HALL P, FLANDERS KC, SPORN M AND MOORGHEN M. (1993). TGFβ expression in the human colon: differential immunostaining of the crypt epithelium. *Br. J. Cancer*, **68,** 137–139.

BAIGRIE RJ AND BERRY AR. (1994). Management of advanced rectal cancer. *Br. J. Surg*., **81,** 343–352.

BARCELLOS-HOFF MH, DERYNCK R, TSANG ML AND WEATHERBEE JA. (1994). Transforming growth factor-beta activation in irradiated murine mammary gland. *J. Clin. Invest*., **93,** 892–899.

CHAKRABARTY S, FAN D AND VARANI J. (1990). Modulation of differentiation and proliferation in human colon carcinoma cells by TGFβ1 and TGFβ2. *Int. J. Cancer*, **46,** 493–499.

CHEN RH, EBNER R AND DERYNCK R. (1993). Inactivation of the type II receptor reveals two receptor pathways for the diverse TGFβ activities. *Science*, **260,** 1335.

COFFEY RJ, SHIPLEY GD AND MOSES HL. (1986). Production of TGFβ by human colon cancer cell lines. *Cancer Res*., **46,** 1164–1169.

FRIESS H, YAMANAKA Y, BUCHLER M, EBERT M, BEGER HG, GOLD LI AND KORC M. (1993). Enhanced expression of TGFβ isoforms in pancreatic cancer correlates with decreased survival. *Gastroenterology*, **105,** 1846–1856.

GEISER AG, BURMESTER JK, WEBBINK R, ROBERTS AB AND SPORN MB. (1992). Inhibition of growth by TGFβ following fusion of two non-responsive human carcinoma cell lines. *J. Biol. Chem*., **267,** 2588–2593.

GORSCH SM, MEMOLI VA, STUKEL TA, GOLD LI AND ARRICK BA. (1992). Immunohistochemical staining for TGFβ1 associates with disease progression in human breast cancer. *Cancer Res*., **52,** 6949–6952.

HARPEL JG, METZ CN, KOJIMA S AND RIFKIN DB. (1992). Control of TGFβ activity: latency *vs* activation. *Prog. Growth Factor Res*., **4,** 321–335.

HIRAYAMA D, FUJIMORI T, SATONAKA K, NAKAMURA T, KITAZAWA S, HORIO M, MAEDA S AND NAGASAKO K. (1992). Immunohistochemical study of EGF and TGFβ in the penetrating type of early gastric cancer. *Hum. Pathol*., **23,** 681–686.

JASANI B, WYLLIE FS, WRIGHT PA, LEMOINE NR, WILLIAMS ED AND WYNFORD-THOMAS D. (1990). Immunocytochemically detectable TGFβ associated with malignancy in thyroid epithelial neoplasia. *Growth Factors*, **2,** 149–155.

JASS JR. (1989). Prognostic variables in large bowel cancer. *J. Clin. Pathol*., **42,** 1006–1007.

KHAN S, RAZA A, PETRELLI N AND MITTLEMAN A. (1988). *In vivo* determinations of labelling index of metastatic colorectal carcinoma and normal mucosa using intravenous infusion of bromodeoxyuridine. *J. Surg. Oncol.*, **39**, 114–118.

KOHNEWOMPNER CH, SCHOFFSKI P AND SCHMOLL HJ. (1994). Adjuvant therapy for colon adenocarcinoma–current status of clinical investigation. *Ann. Oncol.*, **5**, S97–S104.

LAHM H AND ODARTCHENKO N. (1993). Role of TGFβ in colorectal cancer. *Growth Factors*, **9**, 1–9.

LAROCCA RV, PARK JG, DANESI R, DELTACCA M, STEINBERG SM AND GAZDAR AF. (1992). Pattern of growth factor, protooncogene and CEA gene expression in human colorectal carcinoma cell lines. *Oncology*, **49**, 209–214.

MCCARTNEY-FRANCIS NL AND WAHL SM. (1994). TGFβ: a matter of life and death. *J. Leuk. Biol.*, **55**, 401–409.

MANNING AM, WILLIAMS AC, GAME SM AND PARASKEVA C. (1991). Differential sensitivity of human colonic adenoma and carcinoma cells to TGFβ. *Oncogene*, **6**, 1471–1476.

MARKOWITZ S, WANG J, MYENOFF L, PARSONS R, SUN LZ, LUTTERBAUGH J, FAN RS, ZBOROWSKA E, KINZLER KW, VOGELSTEIN B, BRATTAIN M AND WILLSON JKV. (1995). Inactivation of the type II TGFβ receptor in colon cancer cells with microsatellite instability. *Science*, **268**, 1336–1338.

MASSAGUE J, CHIEFETZ S, LAIHO M, RALPH DA, WEIS FMB AND ZENTELLA A. (1992). Transforming growth factorβ. *Cancer Surveys*, **12**, 81–103.

MIYAZONO K, DIJKE PT, ICHIJO H AND HELDIN CH. (1994). Receptors for TGFβ. *Adv. Immunol.*, **55**, 181–221.

MULDER KM, LEVINE AE, HERNANDEZ X, MCKNIGHT MK, BRATTAIN DE AND BRATTAIN MG. (1988). Modulation of cmyc by TGFβ in human colon carcinoma cells. *Biochem. Biophys. Res. Commun.*, **150**, 711–716.

MURTHY U, ANZANO MA AND GRIEG RG. (1989). Expression of TGFαEGF and TGFβ receptors in human colon carcinoma cell lines. *Int. J. Cancer*. **44**, 110–115.

O'CONNELL MJ AND GUNDERSON LL. (1992). Adjuvant therapy for carcinoma of the rectum. *World J. Surg.*, **16**, 510–515.

ODEKON LE, BLASI F AND RIFKIN DB. (1994). Requirement for receptor bound urokinase in plasmin-dependent cellular conversion of latent TGFβ to TGFβ. *J. Cell Physiol.*, **158**, 398–407.

OSAMURA RJ, ODA K, HORI S, TAUCHI K, TOKUDA Y, KUBOTA M AND TAJIMA T. (1990). Evaluation of immunohistochemical expression of TGFβ as a prognostic factor for mammary carcinomas. *Lab. Invest.*, **62**, A76.

ROTHENBERGER DA. (1993). Relevant clinical information and tumour markers. *Int. Cancer*, **71**, 4193–4197.

SCHMOLL HJ. (1994). Colorectal carcinoma–current problems and future perspectives. *Ann. Oncol.*, **5**, S115–S121.

SCHOFIELD PE, WALSH S AND TWEEDLE DEF. (1986). Survival after treatment of carcinoma of the rectum. *Br. Med. J.*, **293**, 496–497.

STEINER MS AND BARRACK ER. (1992). TGFβ1 overproduction in prostate cancer: effects on growth *in vivo* and *in vitro*. *Mol. Endocrinol.*, **6**, 15–25.

THOMPSON TC, TRUONG LD, TIMME TL, KADMON D, MCKUNE BK, FLANDERS KC, SCARDINO PT AND PARK SH. (1992). TGFβ1 as a biomarker for prostate cancer. *J. Cell Biochem.*, **16H**, 54–61.

WAKEFIELD LM, SMITH DM, BROZ S, JACKSON M, LEVINSON AD AND SPORN MB. (1989). Recombinant TGFβ1 synthesized as a two component latent complex that share some structural features with the native platelet latent TGFβ1 complex. *Growth Factors*, **1**, 203–218.

WALKER RA AND DEARING SJ. (1992). TGFβ1 in ductal carcinoma *in situ* and invasive carcinomas of the breast. *Eur. J. Cancer*, **28A**, 641–644.

WOLMARK N, ROCKETTE H, FISHER B, WICKERHAM DL, REDMOND C, FISHER ER, JONES J, MAMOUNAS EP, ORE L, PETRELLI NJ, SPURR CL, DIMITROV N, ROMOND EH, SUTHERLAND CM, KARDINAL CG, DEFUSCO PA AND JOCHIMSEN P. (1993). The benefit of leucovorin-modulated fluorouracil as post-operative adjuvant therapy for primary colon cancer. *J. Clin. Oncol.*, **11**, 1879–1887.